# Bearing dislocation of mobile bearing unicompartmental knee arthroplasty in East Asian countries: a systematic review with meta-analysis

**DOI:** 10.1186/s13018-020-02190-8

**Published:** 2021-01-07

**Authors:** Xiaowei Sun, Pei Liu, Feifan Lu, Weiguo Wang, Wanshou Guo, Qidong Zhang

**Affiliations:** 1 Graduate School of Peking Union Medical College and Chinese Academy of Medical Sciences, Beijing, China; 2 Department of Orthopaedic Surgery, China-Japan Friendship Hospital, No. 2, Yinghuadong Road, Chaoyang District, Beijing, 100029 China; 3 Beijing University of Chinese Medicine, Beijing, China; 4 China-Japan Friendship School of Clinical Medicine, Peking University, Beijing, 100029 China

**Keywords:** UKA, Bearing dislocation, East Asia, Meta-analysis

## Abstract

**Background:**

Bearing dislocation is a common postoperative complication of mobile-bearing unicompartmental knee arthroplasty, and East Asian patients tend to be at higher risk. However, whether this high dislocation rate is common in all East Asian populations remains unclear. This meta-analysis was performed to explore this issue and describe various features of dislocation in East Asians, including the onset time, dislocation direction, and re-dislocation rate.

**Methods:**

The literature was searched in PubMed, Embase, Ovid, and Cochrane Library up to May 2020. Studies were scrutinized by two independent authors, and the bearing dislocation rate, onset time, direction, and re-dislocation rate were specifically analyzed. RevMan 5.3 was used for the statistical analysis.

**Results:**

Seven case series from Korea, China, and Japan were included. The pooled analysis showed that the total dislocation rate was 2.37%, while the subgroup analysis showed that the dislocation rate in Korea and other countries was 4.50% and 0.74%, respectively (*P* < 0.01). Another subgroup analysis of the onset time showed a significant difference before and after the first 5 years postoperatively (*P* < 0.01). Anterior and posterior dislocations were more frequent than medial and lateral dislocations (*P* < 0.01). The average re-dislocation rate was 32.45%, which was approximately seven times higher than the primary dislocation rate (*P* < 0.01).

**Conclusion:**

Our meta-analysis demonstrated that Korea had a higher bearing dislocation rate among East Asian countries, especially in the first 5 years after primary UKA. Anterior and posterior dislocations were common. The most important finding is that the re-dislocation rate can be much higher than the initial dislocation rate.

## Introduction

Unicompartmental knee arthroplasty (UKA) is an effective treatment for patients with unicompartmental knee osteoarthritis. Compared with total knee arthroplasty (TKA), patients who have undergone UKA have better functional recovery but a higher revision rate due to prosthesis failures, among which dislocation of the mobile bearing is considered the main cause [1–3]. In a recently published systematic review of Oxford Phase III UKA, Ro et al. [4] found that Asian patients had a higher dislocation rate than Western patients because of their traditional lifestyle and religious practices, which involve the sitting kneel position or cross-leg sitting. However, whether this fact applies to all East Asian populations remains debatable [4–9]. In this review, we focused only on patients from East Asia who shared similar lifestyle practices involving regular knee hyperflexion to identify differences among countries. Another unresolved issue of concern to orthopedic surgeons is the cause of bearing dislocation. Some researchers have recently suggested that bearing dislocation usually happens shortly after primary UKA because of errors in this procedure [4, 5, 10]. In contrast, some other researchers have shown that bearing dislocation can occur long after the primary operation, indicating that the direct cause might be bearing wear [6, 11]. Because few studies have investigated the mechanism of bearing dislocation, which is even more difficult to review by a meta-analysis, we evaluated the time interval between dislocation and the primary operation to accumulate more evidence. The treatment of bearing dislocation is another important issue for both joint surgeons and patients, and few studies have specifically focused on treatment. In this review, we also focused on the treatment strategy and outcome, especially with respect to the risk of re-dislocation.

The major aim of this meta-analysis was to determine whether the dislocation rates in East Asian countries were different. Second, we explored the epidemiology of this complication and aimed to determine the highest risk time period and most likely direction of dislocation in the Asian population. Third, we sought to identify the most suitable treatment and assessed the re-dislocation rate.

## Material and methods

This systematic review and meta-analysis was performed according to the Preferred Reporting Items for Systematic Reviews and Meta-Analyses (PRISMA) checklist. Ethical approval was not required.

### Literature search

We searched all articles in PubMed, Embase, and Cochrane Library up to May 2020. We also searched Ovid to identify articles that might have been omitted from PubMed. In addition, a manual search of bibliographies of identified articles was performed to identify potentially relevant studies. The search strategy was based on the following keywords: (((UKA OR UKR OR UCR OR unicompartmental knee replacement OR unicompartmental knee arthroplasty OR unicondylar replacement OR unicondylar arthroplasty)) AND (dislocation OR luxation OR malposition)) AND (bearing OR insert OR mensic$ bearing OR mensic$ insert OR mensic$ prosthesis). Our search was not limited to any specific languages, but we restricted the publication time to 20 years because the Oxford Phase III prosthesis was first introduced in 1998.

### Study selection

The study selection criteria were established according to the PICOS strategy (Patients, Intervention, Comparison, Outcomes, Study design): population—East Asian patients who underwent UKA; intervention—UKA with Oxford Phase III prosthesis; comparison—none; outcomes—complications of bearing dislocation with detailed information, including onset time, dislocation direction, and treatment; study design—case series or consecutive case-control studies. Studies were ruled out if they met any of the following exclusion criteria: (1) did not clearly report the reoperation methods; (2) were biomechanical, cadaveric, or radiologic variable measurement studies; (3) were case reports, review articles, meta-analyses, non-original studies, scientific conference abstracts, or other unpublished work; (4) involved fixed UKA or lateral UKA; (5) or were non-consecutive case series. We included only studies involving East Asian patients because these patients are anatomically very similar and share the same lifestyle and religious practices, while studies involving West Asian patients were excluded because these patients less frequently engage in the squat and sitting kneel positions.

### Data extraction

Data extraction was carried out by two independent authors (XW.S. and P.L.) using a predefined data extraction form that was divided into three parts: (1) basic information of each study, including the authors’ names, publication year, name and region of the involved medical center, and total cases of primary UKA; (2) information of each case of dislocation, including the onset time, dislocation direction, reoperation method, and reoperation outcome; and (3) heterogeneity factors including age, sex, body mass index, and follow-up period. For studies in which data were incomplete or unclear, attempts were made to contact the authors for details. All data were extracted, and any disagreement was checked by a senior surgeon and third author (QD.Z.) to reach a final decision.

### Quality appraisal

The modified Coleman methodology score (MCMS) has been used in multiple meta-analyses [4, 12] to evaluate the risk of bias in studies of knee arthroplasty. The total score is 100 points. A score of > 85 is considered “excellent,” 84 to 70 is considered “good,” 69 to 50 is considered “moderate,” and < 50 is considered “poor.” Although all studies were case series with an evidence level of IV, the mean MCMS was 75.4 ± 4.7, which was regarded as good quality. The patients’ details and MCMS of each study are shown in Table 1.

**Table 1:** Characteristics of included studies and MCMS

	Country	Sample duration	Sample size	Dislocation cases	Gender (M/F)	Age (year)	BMI (kg/m^**2**^)	Disease		Follow-up time (year)	MCMS
								OA	SONK		
**1.Bae et al.** [5]*	Korea	2002–2016	1853	67	5/62	64 (48–80)	27 (19–35)	60	7	NA	67
**2.Choy et al.** [11]	Korea	2002–2005	164	15	14/133	65.5 (44–75)	NA	146	18	12.1 (10.1–14)	72
**3.Kang et al.** [6]	Korea	2003–2016	531	22	49/482	67.7 (56-78)	25 (20–29)	531	0	9.6 (6.6–12.5)	75
**4.Kim et al.** [13]	Korea	2002–2015	1410	42	151/1425	62 (43–86)	NA	1410	0	7.17 (0.5–14.42)	78
**5.Tian et al.** [7]	China	2006–2010	440	4	177/263	58.3 (44-81)	24 (23–32)	427	13	6.1 (5–9.3)	80
**6.Xue et al.** [8]	China	2005–2017	708	3	295/339	67.8	31	667	41	6.2 (2.7–12)	80
**7.Yoshida et al.** [9]	Japan	2002–2011	1251	10	180/810	77.2 (47–94)	NA	1186	65	5.2 (1–10.5)	76

*Bae et al. [5] provided basic data of patients with bearing dislocations only

Another method that is used to evaluate the quality and risk of bias is the methodological index for evaluation of non-randomized studies (MINORS) [14]. Several meta-analyses of UKA [10, 15] have used the MINORS with an additional scoring system based on the outcome reporting of primary and revision UKAs. We modified the additional scoring system to suit our study purpose, including criteria of the total number of UKA cases, survival outcome, number of dislocation cases, and revision outcome. We defined > 200 total UKA cases as level “A” because the bearing dislocation rate among Asian patients is approximately 0.53% [4]. We also defined > 10 dislocation cases as level “A.” Moreover, clear statements of the survival outcome of primary UKA and revision of dislocation cases were also considered level “A.” Similar to the studies by Mohammad et al. [10] and Campi et al. [15], we considered studies with a MINORS score of > 80% to have a low risk of bias and those with a MINORS score of < 70% to have a high risk of bias, except studies with three or more level “A”s in an additional diagram. Details are shown in Table 2.

**Table 2:** Modified MINORS scale of risk bias

	MINORS	Total UKA cases	Survival outcome of UKA	Dislocation cases	revision outcome	Risk of bias
**1.Bae et al.** [5]*	12/16	A	A	A	A	Low
**2.Choy et al.** [11]	11/16	B	A	A	A	Low
**3.Kang et al.** [6]	12/16	A	A	A	A	Low
**4.Kim et al.** [13]	10/16	A	A	A	A	Low
**5.Tian et al.** [7]	9/16	A	A	B	B	High
**6.Xue et al.** [8]	10/16	A	A	B	B	Low
**7.Yoshida et al.** [9]	10/16	A	A	A	B	Low

### Statistical analysis

Using RevMan 5.3, the results of the selected studies were pooled for the meta-analysis when two or more results were available. The incidence of bearing dislocation (*p*) was calculated by the total UKAs (*n*) and the cases of dislocation (*x*) (if *np* > 5 and *n*(1 − *p*) > 5, then *p* = *x*/*n*, SE(*p*)=$\sqrt{p\ \left(1-p\right)/n}$; if *np* < 5 or *n*(1 − *p*) < 5, then *p* =  *ln* (*x*/(*n* − *x*)), SE(*p*)=$\sqrt{1/x+1/\left(n-x\right)}$). The same method was applied to the analysis of re-dislocation and the primary dislocation rate. The heterogeneity of the included studies was evaluated by the Q statistic, τ^2^ statistic, and *I*^2^ statistic. A fixed-effects model was used if *P* > 0.1 and *I*^2^ < 50%; otherwise, a random-effects model was used (*P* < 0.1 and *I*^2^ > 50%) [12, 16]. Dichotomous data are described using risk ratios and 95% confidence intervals (CIs). The level of statistical significance was established at *P* < 0.05. The Q test and chi-square test were used to estimate statistical heterogeneity with the *P* value and *I*^2^ statistic; *I*^2^ > 50% was considered to indicate high heterogeneity. Publication bias was evaluated with a funnel plot. All results are presented in the form of forest plots, and *P* < 0.05 was considered statistically significant.

## Results

### Search results

**Fig. 1:**
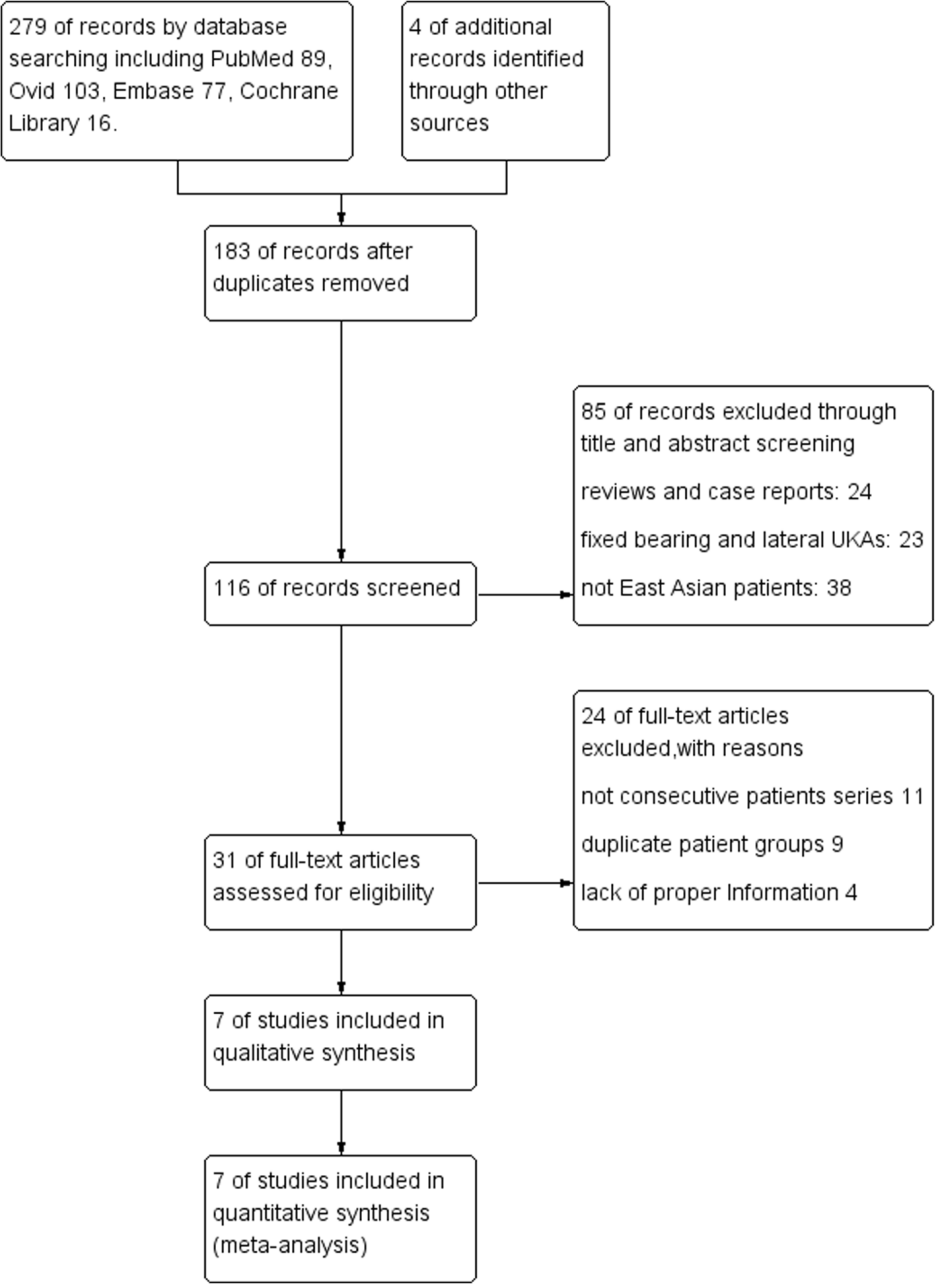
Flow diagram of literature search with inclusion and exclusion criteria

In our search of all databases, we identified 279 articles and removed 183 duplicated records. After reviewing the titles and abstracts, we removed 85 articles and abandoned another 24 after reading the full article because they met the criteria listed in the flow diagram (Fig. 1). In particular, one multicenter study [5] involved a patient series from eight Korean hospitals. After we scrutinized this article along with other studies including the same group of patients [17–20], we decided to include this multicenter study because of its large sample size and detailed information on the outcomes of bearing dislocation management. Finally, we identified seven articles: four from Korea [5, 6, 11, 13], two from China [7, 8], and one from Japan [9].

### Incidence of bearing dislocation

**Fig. 2:**
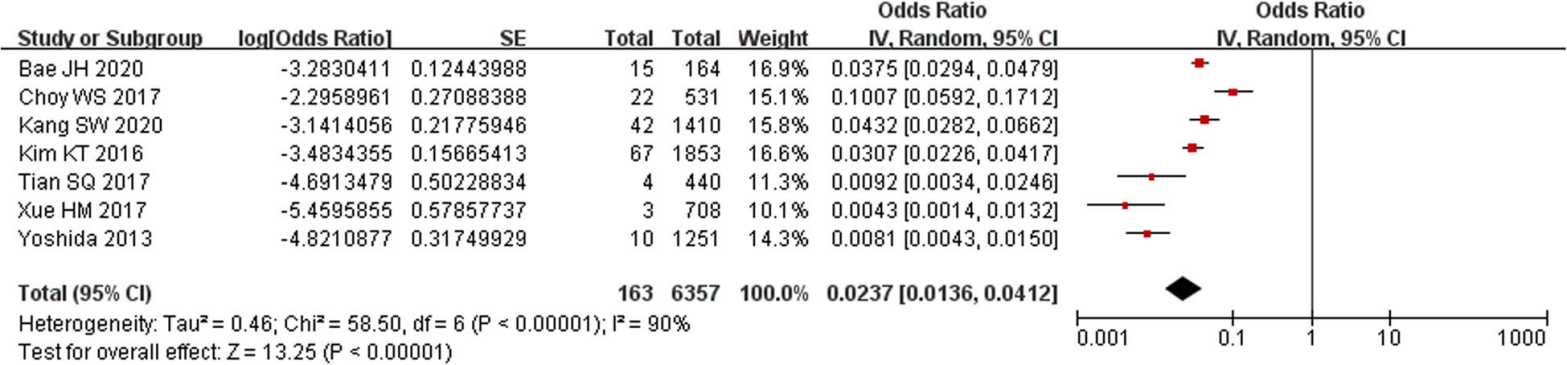
Forest plot of bearing dislocation rate among East Asian patients

Our analysis showed that the bearing dislocation rate among East Asian patients was 2.37% (95% CI, 1.36–4.12%), as shown in Fig. 2. However, the heterogeneity was high as *I*^2^ = 90%.

### Bearing dislocation rate in different countries

**Fig. 3:**
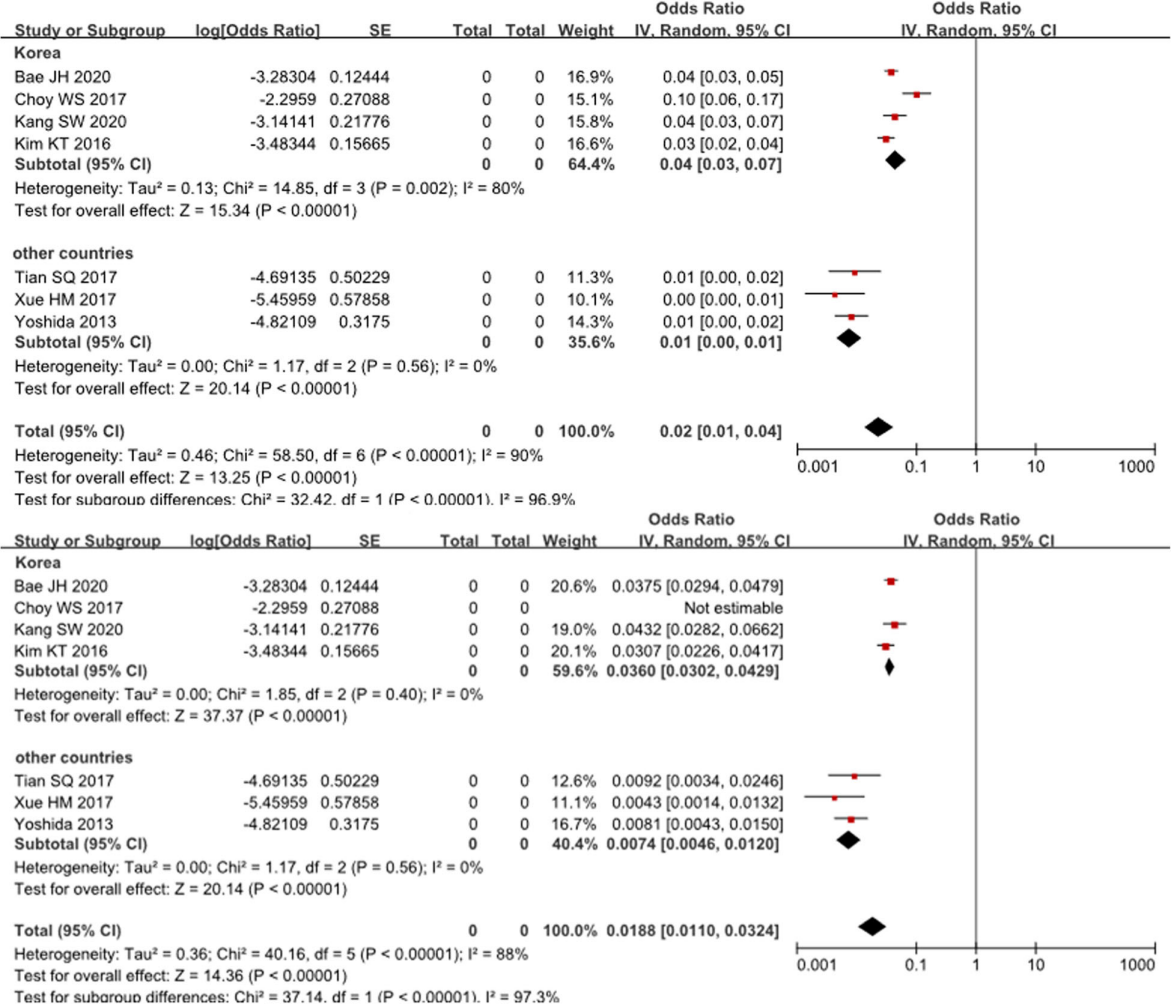
Forest plot of subgroup meta-analysis of Korea and other East Asian countries. (Above) The heterogeneity of the Korean subgroup was high (*I*^2^ = 90%). (Below) When the study by Choy et al. was excluded, the heterogeneity significantly decreased (*I*^2^ = 0%)

In the subgroup meta-analysis, the Korean group had a dislocation rate of 4.50% (95% CI, 3.03–6.68%), which was much higher than that of other countries (0.74%; 95% CI, 0.46–1.20%). As shown in Fig. 3, this difference was statistically significant (*P* < 0.05); however, the heterogeneity within the Korean group was high (*I*^2^ > 50%). We removed each of the four studies one at a time to identify the source of heterogeneity, and we located the study by Choy et al. [11], which was published in 2017. After removing this study, the *I*^2^ decreased to 0%, indicating a low risk of heterogeneity within the subgroup. However, the dislocation rate was still significantly higher than that in other countries, with a mean of 3.60% (95% CI, 3.02–4.29%; *P* < 0.05). Details are shown in Fig. 3.

### Onset time and dislocation direction

**Fig. 4:**
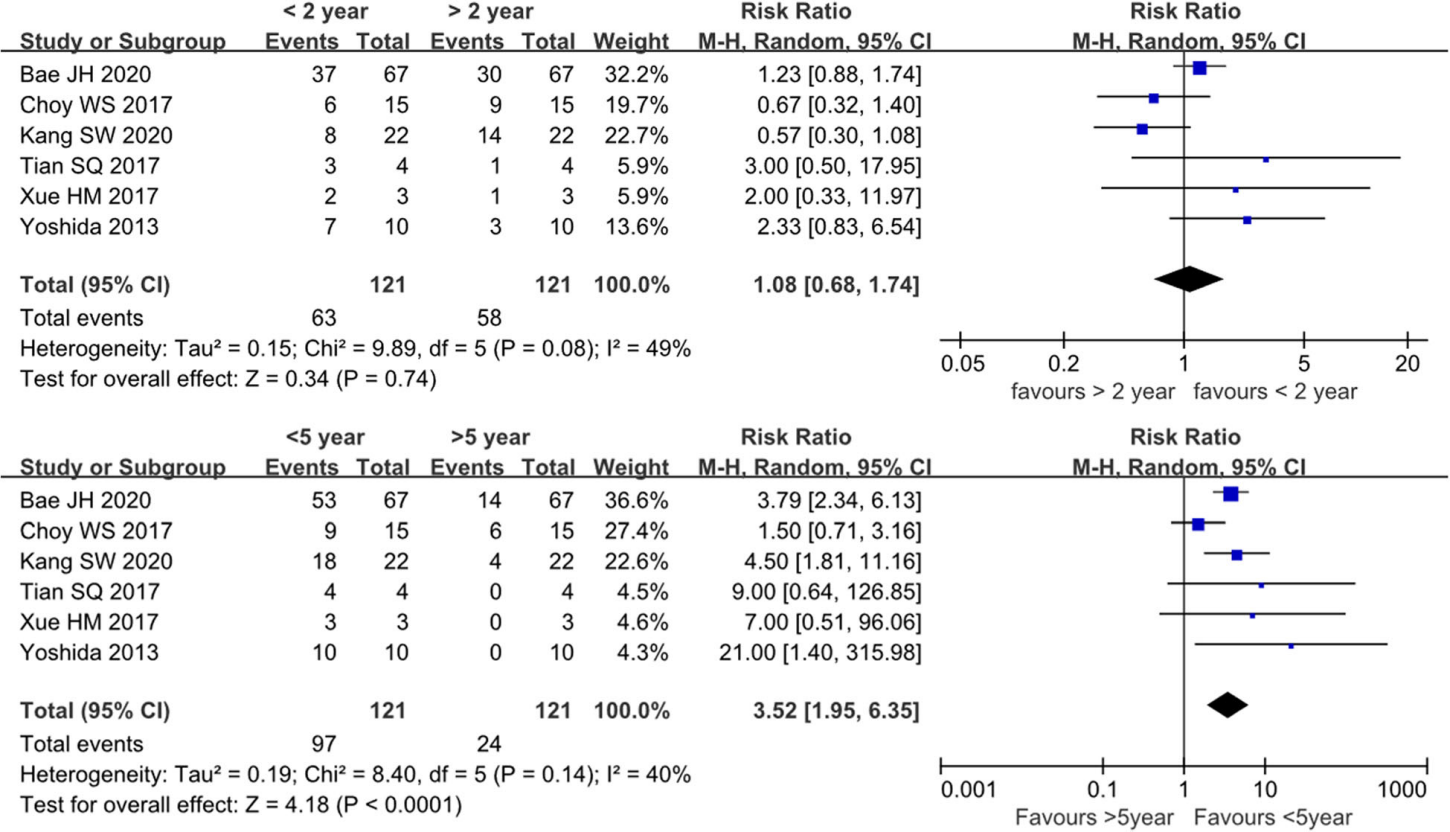
Forest plot of bearing dislocation onset time. (Above) The dislocation rate was not significantly different before and after the first 2 years postoperatively (*P* > 0.05). (Below) The difference in the dislocation rate between the first 5 years and the later phase was statistically significant (*P* < 0.01)

**Fig. 5:**
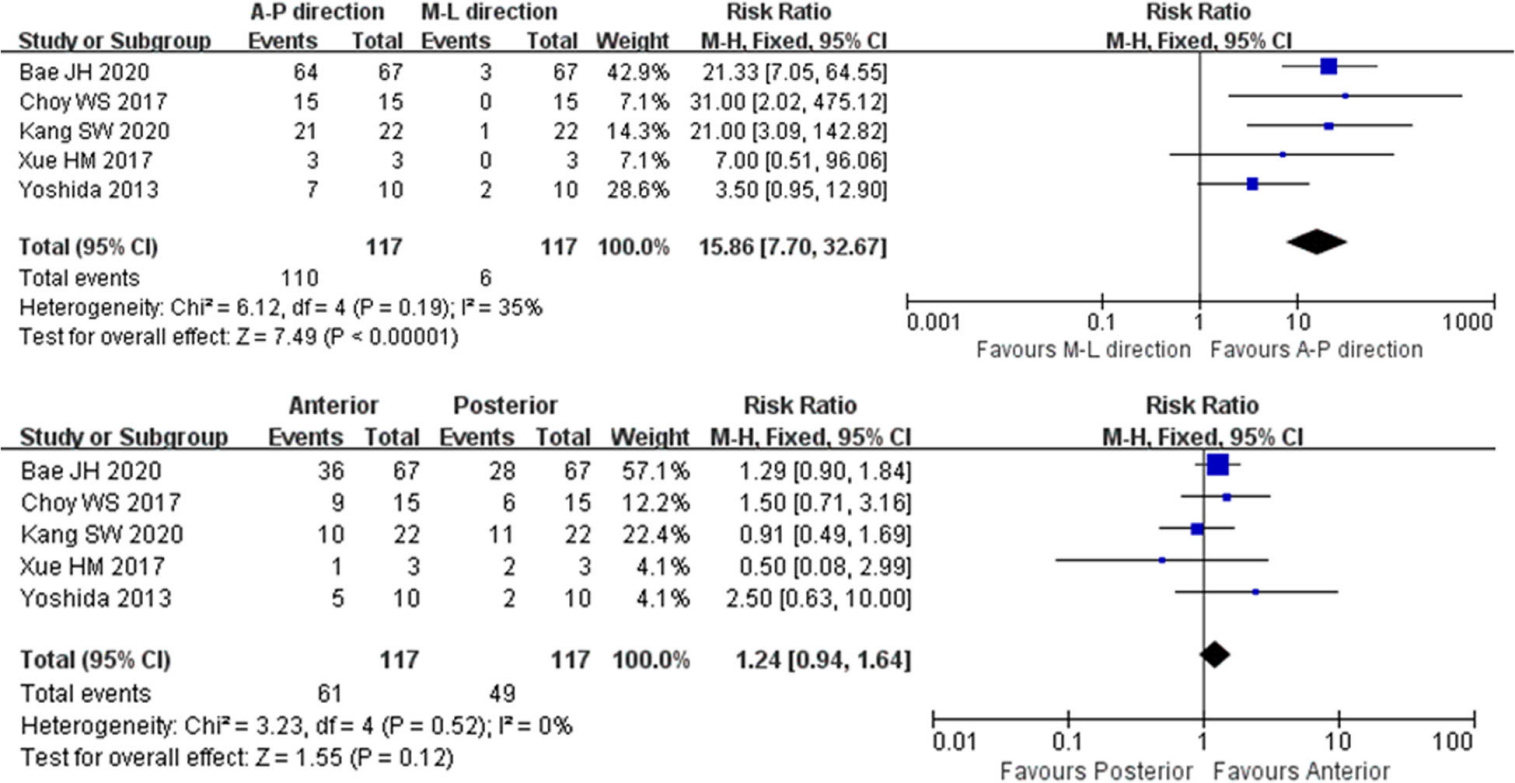
Forest plot of the direction of bearing dislocation. (Above) The difference between anterior-posterior and medial-lateral dislocation was statistically significant (*P* < 0.01). (Below) The difference between anterior dislocation and posterior dislocation was not statistically significant (*P* > 0.05)

Six studies reported the time interval from primary UKA to dislocation. We first applied 2 years as a threshold to divide the early and late stages of bearing dislocation, but the pooled analysis of the onset time showed no difference. However, when we changed the delimitation point to 5 years, the risk of dislocation was significantly different between the two subgroups (*P* < 0.05). Details are shown in Fig. 4. Five studies provided information on the direction of bearing dislocation. The pooled analysis of the direction showed no significant difference between anterior and posterior dislocations (*P* > 0.05); however, they were significantly different from medial and lateral dislocations (*P* < 0.05), which in fact are very rare and seldom seen in case series. Details are shown in Fig. 5 and Table 3.

**Table 3:** Onset time and direction of bearing dislocation

	Total cases	Dislocation time from primary UKA (year)			Dislocation direction			
		< 2	2 to 5	> 5	Anterior	Posterior	Medial	Lateral
Bae et al. [5]	67	37	16	14	36	28	3	0
Choy et al. [11]	15	6	3	6	9	6	0	0
Kang et al. [6]	22	8	10	4	10	11	0	1
Tian SQ et al. [7]	4	3	1	0	NA			
Xue et al. [8]	3	2	1	0	1	2	0	
Yoshida et al. [9]	10	7	3	0	5	2	0	2

### Incidence of bearing re-dislocation

**Fig. 6:**
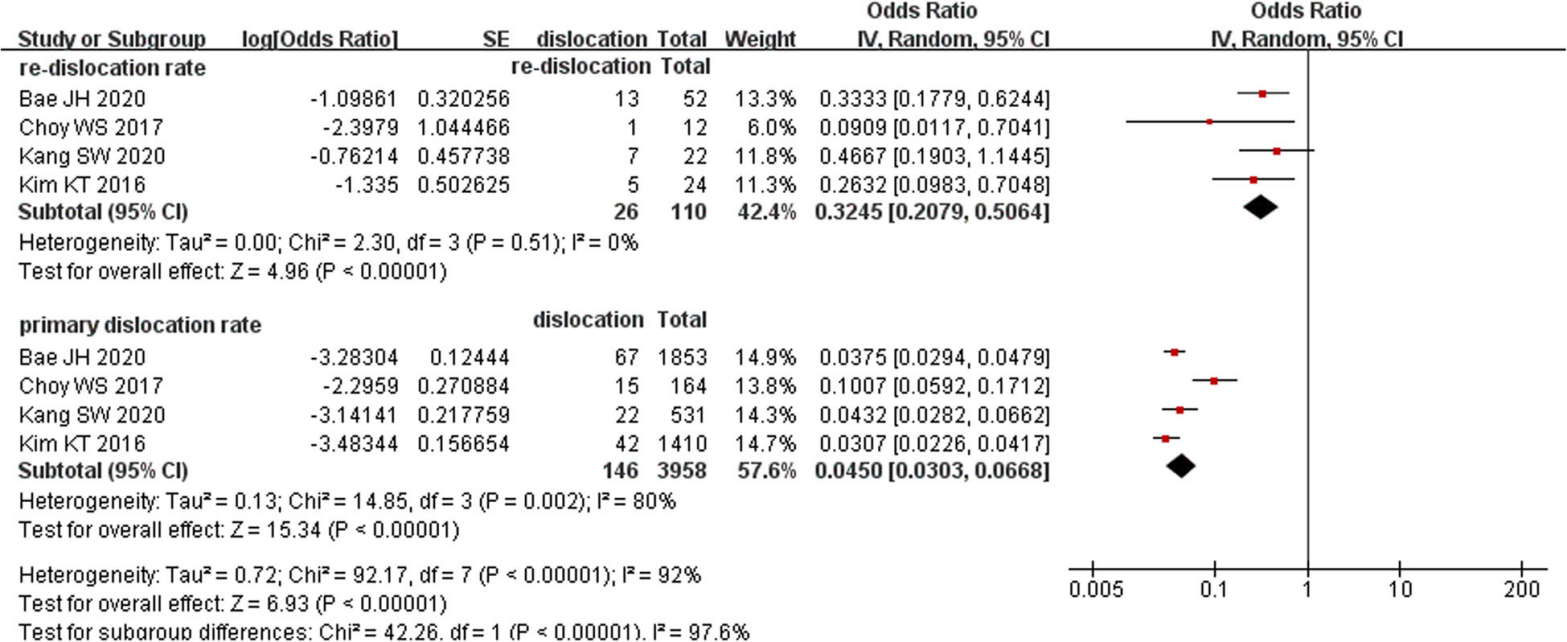
Forest plot of comparison of re-dislocation rate and primary dislocation rate

All four Korean studies reported the treatment outcome of bearing dislocation. Based on this information, we analyzed the re-dislocation rate because it is considered the most common complication of treating dislocations by changing the bearing or performing manipulation under anesthesia (MUA). Unexpectedly, the re-dislocation rate was 32.45% (95% CI, 20.79–50.64%), which was almost seven times higher than the primary dislocation rate (4.50%; 95% CI, 3.03–6.68%; *P* < 0.05). Details are shown in Fig. 6.

### Publication bias

**Fig. 7:**
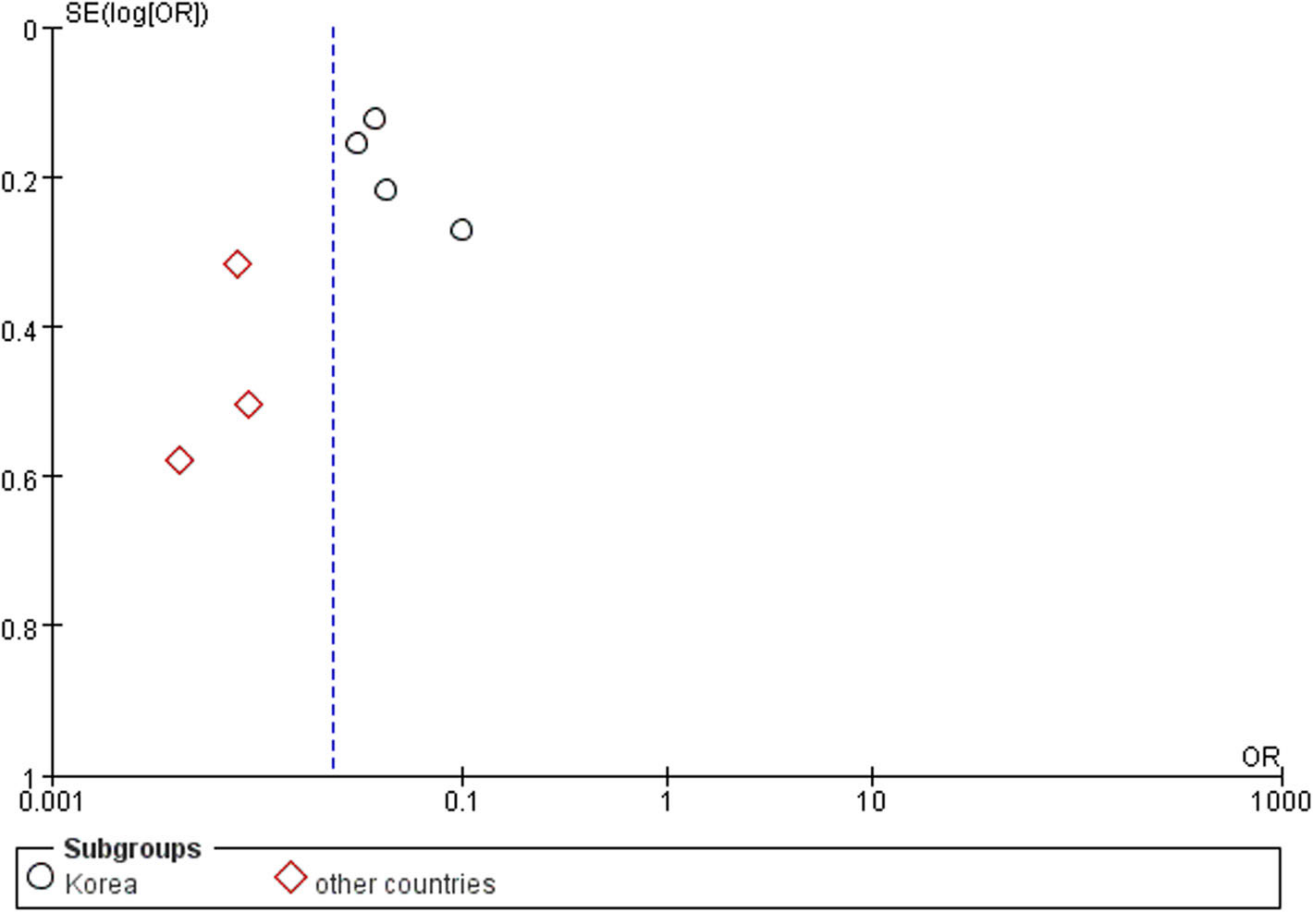
Funnel plot of subgroup meta-analysis between Korea and other East Asian countries

In the funnel plot in Fig. 7, all studies from Korea were located to the right of the baseline, while those from the other East Asian countries lay to the left. This result might indicate that Korean surgeons are more willing to share their failures with the public.

## Discussion

Multiple researchers have agreed that Asian patients have a higher dislocation rate than Western patients after UKA [1, 4–6]. The meta-analysis by Ro et al. [4] showed that the rate was three times higher in East Asian than Western countries and suggested that regular knee hyperflexion along with the anatomical features of the femoral condyle might contribute to this high rate. However, previous research has failed to explore differences among East Asian countries. Although their lifestyle and anatomical structure are similar, Korean patients had an even higher dislocation rate in the present review. In the subgroup of other East Asian countries (China and Japan), the incidence was only 0.74%, similar to the incidence of 0.7% among 1000 cases with a 15-year follow-up as reported by the Oxford group [21]. One possible explanation is that Korean surgeons might be more willing to share their failures with the public; this was also suggested by the funnel plot of publication bias. The high dislocation rate also drew attention, making Korean researchers more willing to take part in the discussion. Another important fact is that there was no artificial joint registry system in any of the East Asian countries, making multicenter studies with large sample sizes difficult to perform. It may be another cause of this difference because a small sample size would lead to selection bias. Nevertheless, the subgroup analysis of other countries suggested that the bearing dislocation rate was not as high as in Korean patients; thus, UKA is still an effective treatment for knee osteoarthritis in East Asian populations.

High heterogeneity was found in the Korean group (*I*^2^ > 50%) when we included the study by Choy et al. [11], which involved 15 dislocations among 164 cases. After removing this study, the *I*^2^ decreased to 0%. This might have been due to the long follow-up period. In their study, Choy et al. [11] evaluated a consecutive UKA case series for an average follow-up of 12.1 years; this is much longer than in the other six reports, in which the follow-up was no longer than 10 years. For this reason, the authors were able to include nine dislocations due to bearing wear, which was considered secondary dislocation and has been seldom seen in other reports. As these late-stage dislocations were added, the dislocation rate became even higher than that in other Korean reports [5, 6, 13]. The results of the present review also highlight the controversy regarding the dislocation onset time. Some researchers believe that most dislocations occur in the early postoperative period and that only a small number of dislocations combined with bearing wear or other complications (periprosthetic fractures, ligament ruptures, or aseptic loosening) occur in the late stage. A newly published multicenter retrospective study [5] showed that most dislocations occurred shortly after surgery, with an incidence of 55% within 2 years and 79% within 5 years. A meta-analysis by van der List et al. [2] also confirmed this. However, when we defined early-stage as 2 years after primary UKA, our meta-analysis showed no difference between these two subgroups. When we adjusted the threshold to 5 years, the difference became statistically significant. This finding might infer that the risk of bearing dislocation is higher during the first 5 years postoperatively. However, because the follow-up time of all included studies was around 6 to 7 years, information on the long-term result of mobile-bearing UKA is limited. Whether the dislocation risk peaks again after 10 years postoperatively remains unknown.

Several reports of the clinical outcomes of UKA have provided information on the direction of bearing dislocation. Most researchers agree that anterior and posterior dislocation is common while medial and lateral dislocation is rare. Our meta-analysis also conformed this, and to our knowledge, it is the first study to apply statistical methods to analyze this. Furthermore, we found no difference between anterior and posterior dislocations, which differs from the findings described in a previous report [22]. This could be explained by determining exactly how bearing dislocation occurs. We explored related research [5, 23–25] of bearing dislocation mechanisms and identified five possible mechanisms: (1) bearing rotation due to malposition of the metallic component, (2) an imbalanced flexion–extension gap, (3) cam impingement, (4) medial collateral ligament (MCL) dysfunction or injury, and (5) an improper bearing size. In fact, these dislocation mechanisms may not exist alone, and the collaboration of multiple mechanisms may underlie the main process of bearing dislocation. The Oxford UKA system is anatomically designed to match the spherical design of the femoral component. The superior surface of the meniscal bearing is concave: thin in the middle with high surrounding rims. The anterior rim is 5 mm, the posterior rim is 3 mm, and medial and lateral rims are both 2 mm. Malposition of either the femoral or tibial component may lead to bearing rotation [25, 26], and in this situation, the lower rim of the medial and lateral part loses its restriction of the mutual movement between the femoral prosthesis and the bearing. As the knee suddenly flexes or extends, the osteophyte in the front or residual meniscus or bone cement in the back may force the bearing out of its normal position toward either the anterior or posterior direction [27]. An imbalanced joint gap, improper bearing size, or luxation of the MCL may all aggravate this phenomenon.

Various treatment options for bearing dislocations are available, including MUA, bearing change, revision to fixed-bearing UKA, and conversion to TKA. UKA metallic component revision has seldom been described in previous reports, possibly because of surgeons’ lack of confidence with this procedure. The treatment information in the articles of the present review is shown in Table 4. In the subgroup meta-analysis of the four Korean reports that provided treatment outcomes, the re-dislocation rate was unexpectedly much higher than the primary dislocation rate. The two most likely causes of this might be as follows. First, the surgeon can remove impingement factors such as osteophytes or redundant cement by exploring the possible causes of dislocation during the revision surgery. However, malposition or poor alignment of the metallic component are beyond the surgeon’s reach in either the bearing change procedure or MUA, in which the newly implanted meniscal bearing is still at high risk of dislocation [22]. Second, potential luxation of the MCL and imbalance of the flexion–extension gap may also contribute. The mechanism of dislocation should be considered before selecting the treatment strategy. Theoretically, re-dislocation will not occur if the underlying causes are corrected during the revision procedure. If not, bearing exchange may not be the most suitable treatment option. Because revision UKA to TKA has been proven effective with a complication rate equivalent to that of primary TKA [28–30], conversion to TKA should definitely be considered.

**Table 4:** Management options of bearing dislocations

	MUA	Bearing change	Fixed UKA	TKA	Total cases
**1.Bae et al.** [5]		52		15	67
**2.Choy et al.** [11]		12		3	15
**3.Kang et al.** [6]	3	19			22
**4.Kim et al.** [13]		24		18	42
**5.Tian et al.** [7]		3		1	4
**6.Xue et al.** [8]		3			3
**7.Yoshida et al.** [9]		2*	3	5	10
**Total**	3	115	3	42	163

*One case was managed by changing meniscal bearing and tibial component

This meta-analysis has four main limitations. First, we included only studies of East Asian patients to determine the difference in the incidence of bearing dislocation among countries. When analyzing the onset time, possible direction, and re-dislocation rate, inevitable selection bias was present because of the exclusion of Western studies. Second, all reports in the present review were case series with an evidence level of IV. Although there were no control groups, the design of the studies was still considered high-quality with a mean MCMS of 75.4 ± 4.7 (considered good quality). The assessment with the modified MINORS score showed that one article from China had a high risk, which may lead to selection bias. Third, the meta-analysis included few case series of UKA complications in China and Japan, which is also a source of bias. Additionally, the follow-up duration in most studies was < 10 years, which may not be long enough to analyze secondary dislocation. As suggested by Choy et al. [11], the timeline of the dislocation risk might have two peaks; however, we only identified the early-stage peak because of the limited follow-up time. Fourth, improvements in the surgical technique and instrumentation of UKA were not described specifically in this meta-analysis. The introduction of Microplasty® instrumentation in 2014 was considered an effective method to decrease the incidence of complications [6, 19, 25]. However, there are still no adequate clinical results available for analysis. During the next few years, we might be able to determine whether this instrument can decrease the dislocation rate by evidence-based methods.

## Conclusion

In conclusion, our meta-analysis has demonstrated that Korea has a higher bearing dislocation rate among East Asian countries. Additionally, mobile bearings have a higher risk of dislocation in the first 5 years after UKA. Anterior and posterior dislocations are most common, but we found no difference between these two directions. The most important finding is that the re-dislocation rate is likely to be much higher than the initial dislocation rate if unsuitable management is applied.

## Data Availability

The datasets generated and/or analyzed during the current study are available from the corresponding author on reasonable request.

## Abbreviations

UKA: Unicompartmental knee arthroplasty
TKA: Total knee arthroplasty
MCMS: Modified Coleman methodology score
MINORS: Methodological index for evaluation of non-randomized studies
CI: Confidence interval
MUA: Manipulation under anesthesia
MCL: Medial collateral ligament
